# Obstructive Sleep Apnea as a comorbidity to Covid-19

**DOI:** 10.5935/1984-0063.20200064

**Published:** 2020

**Authors:** Sergio Tufik

**Affiliations:** Departamento de Psicobiologia, Universidade Federal de São Paulo, São Paulo, SP, Brazil.

Obstructive Sleep Apnea (OSA) is the most common sleep-related breathing disorder being highly prevalent worldwide. It characterizes by transient increases in upper airway resistance, reducing and interrupting the airflow, with elevated respiratory effort. In response to these events, the body becomes hypoxemic and lead to increased oxygen demand, affecting cardiac tissue which can trigger both atrial and ventricular arrhythmias. In addition to hypoxia, OSA induces awakenings during sleep, resulting in sleep deprivation and poor sleep quality. The sleep deprivation is associated with alterations in immune response, in brain neurotransmitter function and in metabolic process of our body^1^.

Covid-19 manifests as respiratory compromise in some patients infected by the disease. It has been observed that this new pathology resulted from SARS-CoV-2 involves respiratory failure, myocardial injury, systemic inflammation, altered myocardial demand-supply balance and electrolyte imbalance^2^. All these events have potential and negative effects in patients who have diabetes, heart diseases and obesity. Studies have confirmed that aged and male patients are also in a greater risk to develop the severe Covid-19. The pathophysiology of Covid-19 has not been well understood. Many studies have been performed to investigate the course of the disease and how SARS-CoV-2 acts in lungs, heart, kidneys, brain and in immune responses. The alterations in body after Covid-19 infection predispose patients to develop severe systemic symptoms, and sometimes death.

It is important to point out the similarities between Covid-19 and OSA in respect to population risk and outcomes^3^. Both diseases are more prevalent in males, aged, obese, diabetics and patients with cardiac injuries. All these comorbidities lead to physiological changes in body function, which may intensify the symptoms and the severity of OSA and Covid-19. The hypoxia presented in OSA may interact with pulmonary parenchymal involvement along with pulmonary vascular endothelial dysfunction from the infectious response to SARS-CoV-2 and can be a potential factor to aggravate the severity and infection in patients with Covid-19. In respect to cardiac outcomes, we should emphasize that OSA is strongly associated with hypertension^4^ and arrhythmias, inducing onset atrial fibrillation and recurrence after different types of antiarrhythmic treatments. Nocturnal cardiac arrhythmias are prevalent in 92.3% of patients with severe OSA and 40% of all patients with OSA^5,6^. Patients with severe Covid-19 infection present cardiac myocyte injury that is suspected to be related to ventricular tachycardia/fibrillation; however, the mechanisms are still under investigation. 44% of patients hospitalized with Covid-19 admitted to intensive care unit presented arrhythmias as demonstrated by a study with 138 patients^2^. In respect to hypertension, 23.7% of patients who developed severe Covid-19 outcomes have hypertension as comorbidity and 16.2% have diabetes^7^.

At this moment, OSA has not been stablished as a risk factor for patients with Covid-19. However, the comorbidities of this sleep disorder, such as diabetes, hypertension, obesity and arrhythmias have been strongly associated with severe Covid-19. Most of patients with OSA have one or more of these comorbidities or will develop if no intervention were required. A factor that has been neglected by researches is the damage in body function caused by sleep deprivation experienced by patients with OSA. It has been demonstrated that sleep deprivation induces immune suppression, which can facilitate susceptibility to SARS-CoV-2 infection. The mechanism involved in the immune response is related with sustained activation of the inflammatory response in persistent sleep disturbances, resulting in chronic inflammation that is recognized to predispose major diseases, such as depression, cancers, cardiovascular diseases, among others^6^. Covid-19 has been attributed to increased inflammatory indicators and the presence of a cytokine storm^2^, as well as sleep disturbances are associated with higher levels of C-reactive protein and interleukin-6^8^. The role of sleep deprivation of OSA becomes significant in respect to infection risk, predicting pneumonia risk and susceptibility to the common cold^9,10^.

Up to this moment, we can conclude that OSA and Covid-19 have similarities and have effects on inflammatory and cardiovascular outcomes^3^. Although OSA has been a common sleep-related breathing disorder^11^, many patients are still underdiagnosed, which can complicate their body response to SARS-Cov-2 infection. Patients with OSA that are in treatment with CPAP should maintain the treatment to avoid/reduce progression of Covid-19 symptoms. In this new scenario of pandemic caused by SARS-CoV-2, we should pay attention to patients with OSA, since they may be more prone to develop the severe degree of Covid-19.

## References

[1] Tufik S, Andersen ML, Bittencourt LRA, Mello MT (2009). Paradoxical sleep deprivation: neurochemical, hormonal and behavioral alterations. Evidence from 30 years of research. Anais da Academia Brasileira de Ciências.

[2] Lazzerini PE, Boutjdir M, Capecchi PL (2020). COVID-19, arrhythmic risk, and inflammation. Circulation.

[3] Tufik S, Gozal D, Ishikura IA, Pires GN, Andersen ML (2020). Does obstructive sleep apnea lead to increased risk of COVID-19 infection and severity?. J Clin Sleep Med.

[4] Cintra FD, Tufik S, de Paola A, Feres MC, Melo-Fujita L, Oliveira W, Rizzi C, Poyares D (2011). Cardiovascular profile in patients with obstructive sleep apnea. Hypertension.

[5] Gami AS, Hodge DO, Herges RM (2007). Obstructive sleep apnea, obesity, and the risk of incident atrial fibrillation. J Am Coll Cardiol.

[6] Cintra FD, Leite RP, Storti LJ (2014). Sleep apnea and nocturnal cardiac arrhythmia: a populational study. Arq Bras Cardiol.

[7] Guan W, Ni Z, Hu Y (2020). Clinical characteristics of coronavirus disease 2019 in China. New England J Med.

[8] Irwin MR (2019). Sleep and inflammation: partners in sickness and in health. Nature Reviews.

[9] Cohen S, Doyle WJ, Alper CM, Janicki-Deverts D, Turner RB (2009). Sleep habits and susceptibility to the common cold. Arch Intern Med.

[10] Prather AA, Janicki-Deverts D, Hall MH, Cohen S (2015). Behaviorally assessed sleep and susceptibility to the common cold. Sleep.

[11] Tufik S, Santos-Silva R, Taddei JA, Bittencourt LRA (2010). Obstructive sleep apnea syndrome in the São Paulo Epidemiologic Sleep Study. Sleep Medicine.

